# The reversed halo sign in pulmonary paracoccidioidomycosis

**DOI:** 10.1590/0037-8682-0330-2025

**Published:** 2025-09-29

**Authors:** Gláucia Zanetti, Elcio Bakowski, Bruno Hochhegger, Edson Marchiori

**Affiliations:** 1 Universidade Federal do Rio de Janeiro, Rio de Janeiro, RJ, Brasil.; 2 Hospital Vivalle, São José dos Campos, SP, Brasil.; 3 University of Florida, Gainesville, Florida, USA.

A 59-year-old non-smoking man with no prior history of rural environmental exposure presented with a 3-month history of dyspnea, persistent nonproductive cough, and progressive clinical deterioration. He reported an 11-kg weight loss over the same period and denied having a fever. Physical examination and laboratory test results were unremarkable.

**FIGURE 1:**
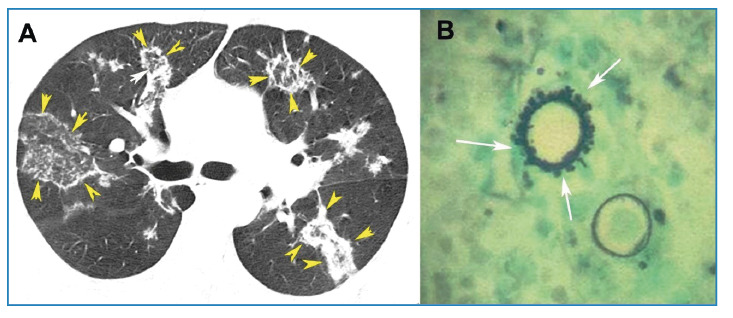
**(A)** Axial CT image at the level of the carina showing bilateral round and oval areas of ground-glass attenuation, most surrounded by rings of consolidation, consistent with the reversed halo sign (yellow arrowheads). **(B)** Fungal organisms with rounded structures and lateral budding forms (arrows), compatible with Paracoccidioides sp. (Grocott stain, 400×).

Chest computed tomography (CT) revealed multiple round and oval focal areas of ground-glass opacity surrounded by rings of consolidation, known as the reversed halo sign (RHS) (Figure 1A). Serological test results were negative. Fiberoptic bronchoscopy with bronchoalveolar lavage, including direct examination and cultures for mycobacteria and fungi, also yielded negative results. A transcutaneous needle biopsy of a lung lesion was subsequently performed, and microscopy revealed typical fungal structures consistent with*Paracoccidioides brasiliensis*(Figure 1B). A final diagnosis of paracoccidioidomycosis (PCM) was established. The patient received antifungal therapy, which resulted in complete resolution of symptoms and marked regression of lung lesions on follow-up CT.

PCM, caused by the dimorphic fungus *P. brasiliensis*, is the most common systemic mycosis in Latin America, particularly in Brazil^1^ ^-^ ^3^. Definitive diagnosis relies on fungal identification through microscopic examination of fresh or biopsy specimens, supplemented by fungal culture and isolation^2^. Chest CT is the preferred imaging modality for assessing pulmonary PCM. In approximately 10% of patients with active infection, it demonstrates the RHS, characterized by a round or oval area of ground-glass opacity completely or partially surrounded by a ring of consolidation in the lung parenchyma^1^ ^-^ ^6^.
